# Recurrent Pneumococcal Meningitis From Post-traumatic Frontoethmoidal Encephalocele: A Case Report

**DOI:** 10.7759/cureus.98862

**Published:** 2025-12-10

**Authors:** Yazan Mazen, Ahmed Darweesh, Maryam Al Ani, Nada Alfalasi, Asif Mohamed Salim, Leena Abdelrahman

**Affiliations:** 1 Medicine, University of Sharjah, Sharjah, ARE; 2 Internal Medicine, Dubai Hospital, Dubai Health, Dubai, ARE

**Keywords:** csf leak, encephalocele, endoscopic repair, recurrent bacterial meningitis, skull defects, streptococcus pneumoniae

## Abstract

Recurrent bacterial meningitis (≥2 episodes with complete recovery between episodes) warrants evaluation for skull-base defects with cerebrospinal fluid (CSF) leak and for immune deficiencies. In adults, *Streptococcus* *pneumoniae* often indicates an anatomic breach (e.g., post-traumatic encephalocele/CSF rhinorrhea), whereas *Neisseria* suggests complement deficiency. We report a case of a 22-year-old man with his second attack of pneumococcal meningitis within a one-year timeframe after sustaining a road traffic accident about four years ago. The patient sustained a left frontal bone fracture communicating with the left frontal sinus, along with a left frontal encephalocele. His current complaint was fever and cough of one week duration; they were associated with headache, rhinorrhea, vomiting, sore throat, fatigue, and generalized body pain. The patient denied having any past history of recurrent or severe infections, especially during childhood and adulthood. Upon assessment, his temperature was 37.6 °C, and his vitals were within normal limits. Physical examination was remarkable for positive neck stiffness. Otherwise, no skin rashes were noted; neurological assessment included a Glasgow Coma Scale of 15/15, no focal neurological deficits, and intact cranial nerves. His abdomen was soft, lax, and non-tender; his spleen was not palpable. Labs were significant for leukocytosis, elevated inflammatory markers, hypoglycorrhachia, and elevated CSF protein. Both blood and CSF cultures were positive for *S.* *pneumoniae*. His HIV test was negative. He was empirically treated with IV ceftriaxone and vancomycin, and later tailored to ceftriaxone after the cultures’ susceptibility results were out. In adults with recurrent pneumococcal meningitis, prompt skull-base imaging for occult CSF leak/encephalocele, and parallel immune evaluation are essential. Definitive endoscopic repair plus pneumococcal vaccination can prevent further episodes. Structured pathways that trigger beta-2 transferrin testing and high-resolution CT/MRI after a second episode may reduce diagnostic delay.

## Introduction

Meningitis is defined as inflammation of the meninges, the layers covering the brain and spinal cord. The most common causes of meningitis are bacterial and viral [1]. Most common causes of bacterial meningitis in patients older than one month of age (i.e., neonates) are *Streptococcus pneumoniae* and *Neisseria meningitidis*, accounting for more than 70% of bacterial meningitis cases in Europe, and 10-20% of the cases in the United States. Both are associated with high mortality rates, with *S. pneumoniae* having a higher rate compared to the latter. *S. pneumoniae* had case fatality rates of 20-37% in high-income countries, versus approximately half of the fatality cases in low-income countries; *N. meningitidis* was associated with 3% in high-income countries, and 10% in low-income countries [2]. In addition, pneumococcal meningitis had an overall higher risk of complications compared to meningococcal meningitis, especially in seizures and hearing loss in affected patients [2].

On the other hand, the definition of recurrent meningitis is controversial. The majority agreed with a definition of two or more episodes of meningitis - caused by different organisms, or the same organism - after at least three weeks of completing therapy [3,4]. Causes of recurrent meningitis can be arranged into two large categories: congenital and acquired. Each can be further subclassified into anatomical anomalies, immunodeficiencies, and chronic parameningeal infections. Overall, anatomic anomalies were attributed as the leading cause, with head injuries and basal skull fractures as the most common anatomical defects noted [4]. A similar pattern was noted by Adriani et al., where remote head injury was reported as the most common predisposing risk factor in the study [5]. *S. pneumoniae* was, by far, the most commonly isolated organism in these studies [3-6].

Encephaloceles - herniation of brain parenchyma through cranial and facial defects - are associated with recurrent meningitis. They are mostly congenital but can also be acquired, usually secondary to head and neck surgeries, head traumas, tumors, or hydrocephalus [4,6,7]. Encephaloceles are estimated to account for 15-20% of neural tube defects [7]. Data on encephaloceles’ incidence and prevalence mostly pertain to the congenital variant; incidence is estimated at one in 10,000 live births. Occipital encephaloceles are the most common subtype (at least 70% of encephaloceles involve the occipital region), whereas frontal subtypes are relatively rare. Variations have been reported depending on geographic locations. Anterior encephaloceles are estimated to occur in one in 35,000 live births in Western populations, but rise to one in 3,500-6,000 live births in Asia, Africa, and Russia; occipital encephaloceles are common in the Western world [7]. This report aims to highlight that an underlying cause should be investigated when a patient presents with recurrent meningitis, through thorough history and physical examination, followed by stepwise investigations. Additionally, this case report aims to improve clinicians’ awareness about recurrent meningitis and encephaloceles and guide the approach to such conditions. This is because missing or neglecting to look into an associated abnormality (e.g., anatomical or immunologic) may lead to further complications other than infection recurrence.

## Case presentation

**Figure 1:**
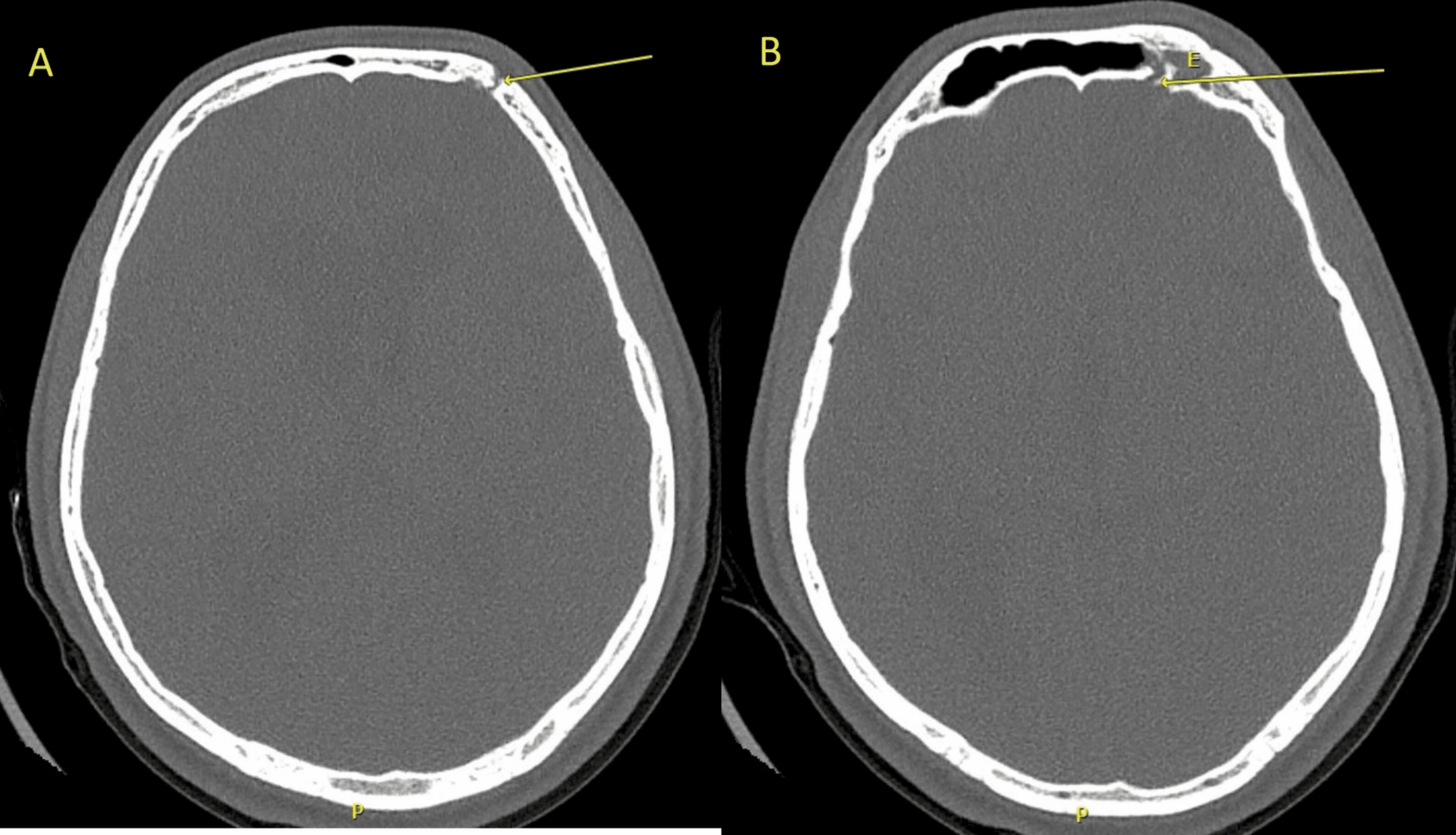
Axial non-contrast cranial CT scan bone window sequence showing the fracture site at the left frontal bone (arrows in sections A and B) with encephalocele (E) protruding the left frontal sinus (in section B).

**Figure 2:**
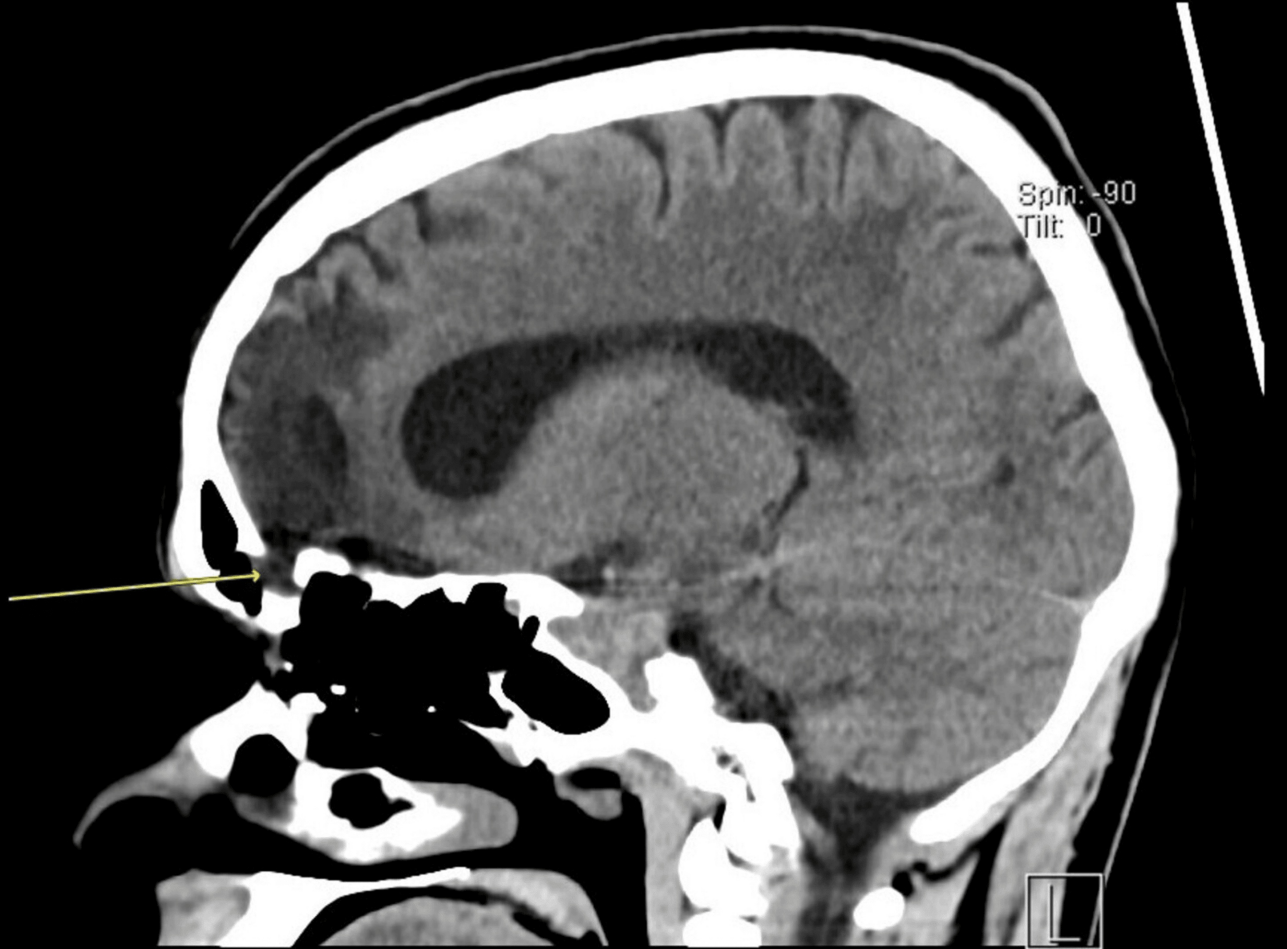
CT scan’s sagittal view showing a communicating defect and an encephalocele (arrow).

A 22-year-old man presented to the emergency department with a fever and a cough of one week's duration. Prior to the current presentation, he had signs and symptoms of upper respiratory tract infection, which manifested as rhinorrhea, sore throat, cough, fatigue, and generalized body pain. His current presentation is also associated with headache, irritability, and vomiting of one day's duration. His medical history is significant for a road traffic accident (RTA) four years back, with imaging revealing a left frontal bone fracture that was communicating with the left frontal sinus. Left-sided frontal encephalocele was noted as well. Last year, he was diagnosed with severe pneumococcal meningitis, which was managed in the intensive care unit due to a decreased Glasgow coma score (GCS) of 11-12/15 for closer observation, and intravenous (IV) ceftriaxone for four weeks due to suspected brain abscess collection, which was excluded later during his admission. Figures 1-2 show the patient’s brain computed tomography (CT) scans without contrast from the previous admission. He refused to undergo surgical repair of the cranial defect and went for conservative management. He also denied having a history of recurrent infections during childhood or prior to the head trauma event.

**Figure 3:**
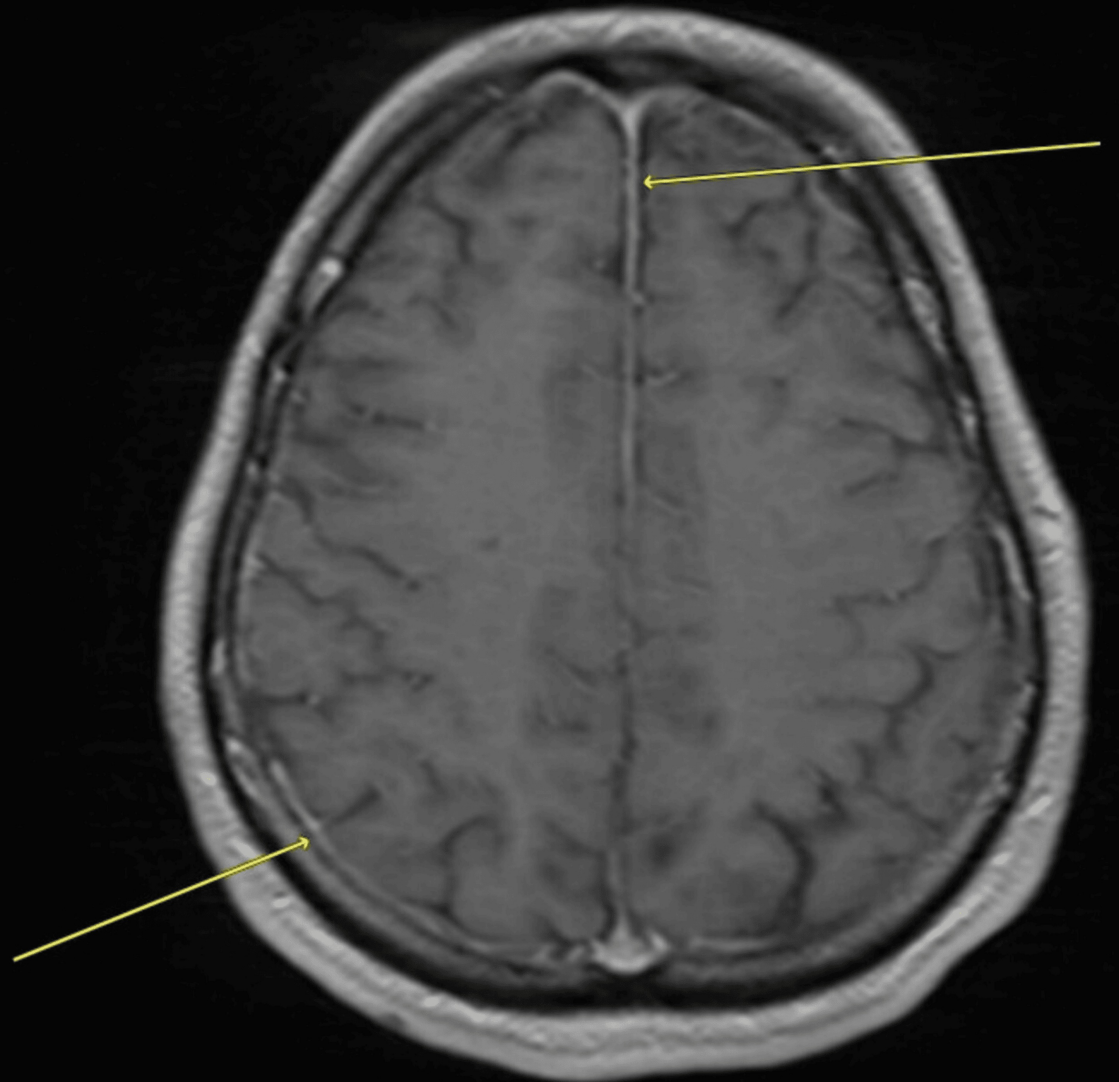
MRI scan’s T1 axial view with contrast showing leptomeningeal enhancement (arrows).

**Figure 4:**
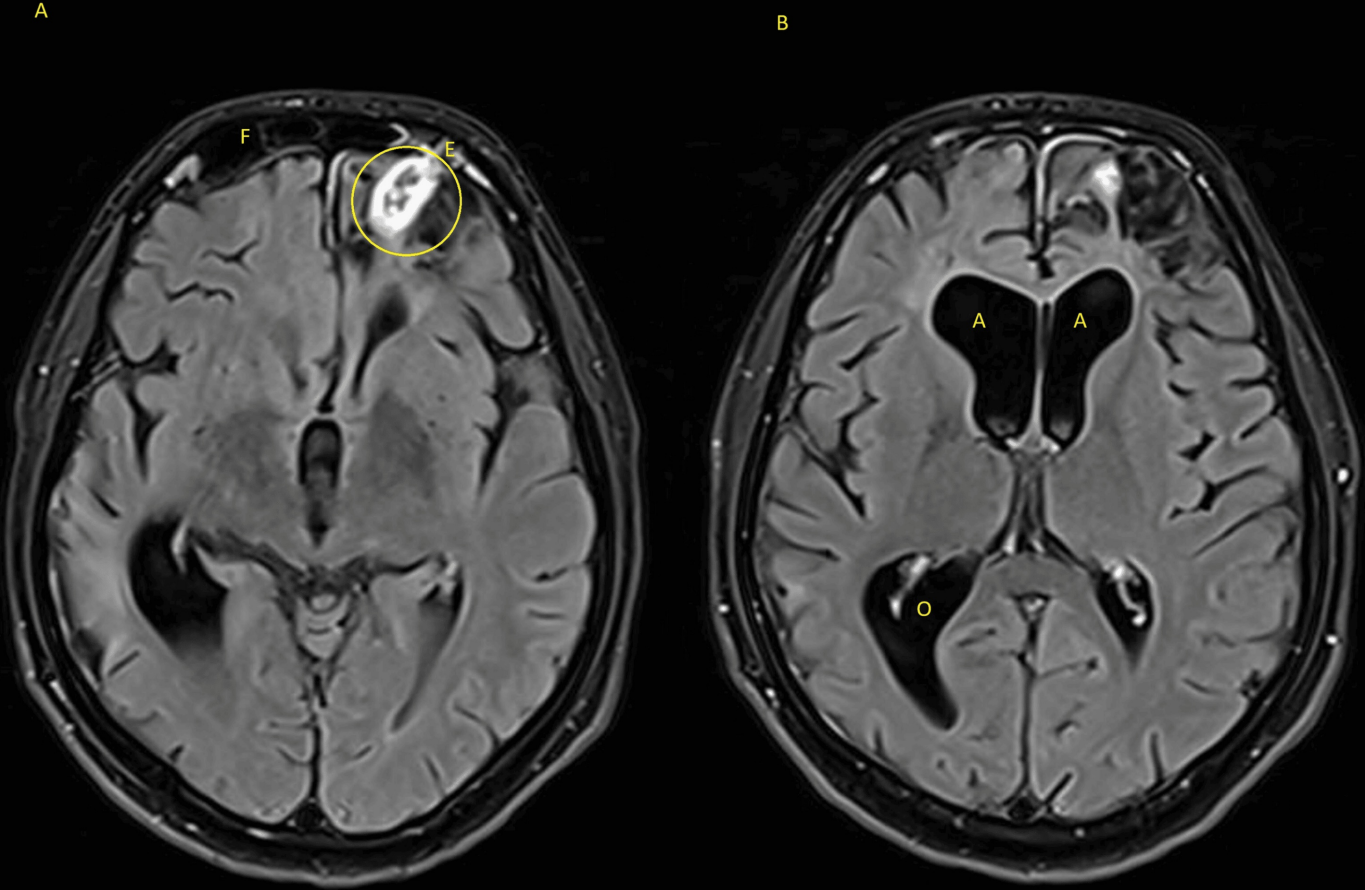
MRI scan’s T1 axial view with contrast showing, in section A, a ring-enhancing collection (circle) in the left frontal lobe, and an encephalocele (E) in the frontal sinus (F). Section B shows hydrocephalus in the anterior (A) and occipital (O) horns of the lateral ventricle.

In the present visit, his temperature was 37.6 °C, and other vital signs were within normal limits. Systemic examination was unremarkable. Neurological examination revealed an intact level of consciousness, no focal neurological deficits, and positive nuchal rigidity. Initial CT scan without contrast revealed hydrocephalus, static changes in the brain parenchyma, abnormalities in the left frontal lobe, and findings suggestive of an inflammatory or abscess collection in the left frontal lobe (close to the encephalocele site). Papilledema was excluded by fundoscopy, and was followed by a lumbar puncture and cerebrospinal fluid (CSF) analysis and culture. Blood tests and culture were obtained as well. The CSF’s appearance was slightly turbid. Table 1 shows the laboratory test results, which were suggestive of a bacterial meningitis pattern, as evidenced by: leukocytosis, elevated inflammatory markers, high CSF protein and low CSF glucose and CSF: serum glucose ratio. *Streptococcus pneumoniae *was isolated from blood and CSF cultures and was identified in the CSF meningitis/encephalitis panel. Acid-fast bacilli smear and culture and fungal cultures were negative. The bacteria were sensitive to cefepime, ceftriaxone, and penicillin G and was resistant to clindamycin and erythromycin. The transthoracic echocardiogram study was normal. The human immunodeficiency virus (HIV) test was negative as well. A brain magnetic resonance imaging (MRI) scan with contrast confirmed the diagnosis of meningitis and revealed that the collection was highly suggestive of an abscess (around 2 x 1 cm in size). Figure 3 shows the meningeal enhancement, and Figure 4 shows the abscess, about 2 x 1 cm in size, adjacent to the encephalocele site and associated hydrocephalus.

**Table 1:** Laboratory test results from the patient’s admission workup. CSF: cerebrospinal fluid

Variable		Result	Reference Range
Serum	WBC count	21.1 x 10^3^ per µL	4-11 x 10^3^ per µL
	CRP	198.7 mg/L	< 3 mg/L
	Lactic acid	3.62 mmol/L	0.5-2.2 mmol/L
	Procalcitonin	0.64 ng/mL	< 0.10 ng/mL
	Glucose, random	146 mg/dL	< 200 mg/dL
CSF	WBC count with differential	3200 cells/µL 85% polymorphonuclear cells 15% lymphocytes	0-5 cells/µL
	Protein	209 mg/dL	15-60 mg/dL
	Glucose	35 mg/dL	50-80 mg/dL
Glucose, CSF:serum ratio		0.24	0.41-0.88

**Figure 5:**
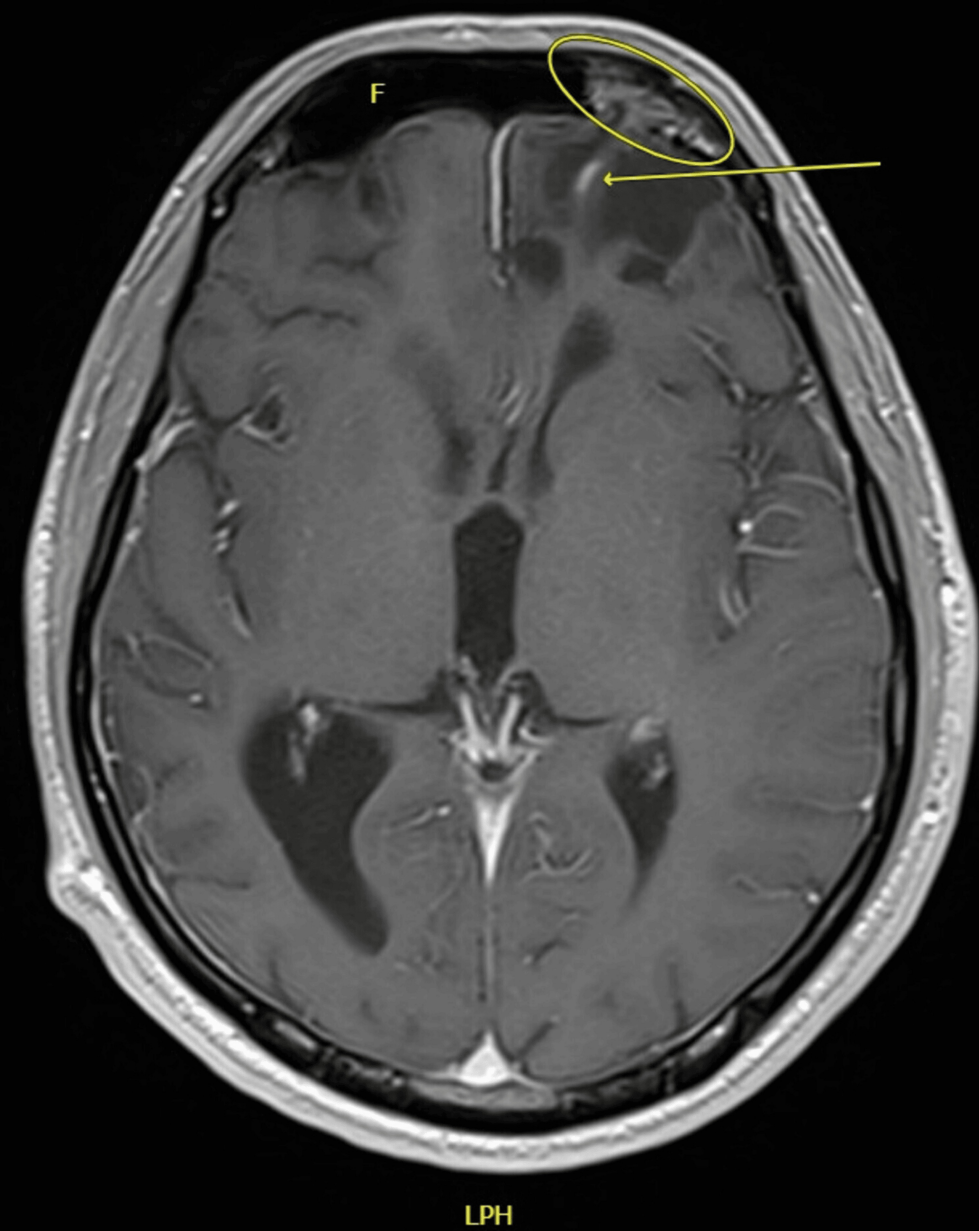
MRI scan’s T1 axial view with contrast showing residual linear peripheral enhancement (arrow) seen at the site of the previously noted left frontal ring-enhancing space-occupying lesion. Encephalocele (eclipse) is still seen in the left frontal sinus (F).

He empirically received IV ceftriaxone 2 g twice daily, IV vancomycin 1,000 mg twice daily for five days, and IV dexamethasone 10 mg for one day. Targeted therapy for *S. pneumoniae *was done using IV ceftriaxone 2 g twice daily, since the patient was already started on ceftriaxone. Although the patient was offered surgery to treat the brain abscess, he opted for medical management. During his inpatient course, his signs and symptoms have improved since starting antibiotics, with fever resolving and WBC count normalizing. His headache has also reduced in severity and did not require intensive care or high dependency units admission - unlike his previous meningitis episode. The repeat MRI image, illustrated in Figure 5, shows a reduction in the size of the abscess, and can be incorporated with Figure 4 as a comparison of the before-and-after treatment.

The infectious disease team was consulted for duration of antibiotics and recommended six weeks of IV ceftriaxone 2 g twice daily to be completed. Blood culture was repeated after antibiotic course completion and was negative. The team also recommended that the patient take pneumococcal and meningococcal vaccines prior to discharge and later from a local primary health center. The intracranial osseous defects were evaluated by otolaryngology and neurosurgery specialists, and surgery was recommended to resolve the likely source of his current and previous infections. However, the patient refused surgery and wanted to consult other physicians - including doctors from his home country - regarding alternative treatments. He attended the neurosurgery follow-up clinic after discharge; he is asymptomatic with no active complaints. He has been free of any recurrence for seven months after discharge.

## Discussion

Encephaloceles are intracranial contents that herniate due to a defect in the cranial vault or base of the skull. They can be acquired as a consequence of trauma, infection, or iatrogenic causes, or congenitally as a result of insufficient closure of the neural tube during development [7]. In this case, the patient has a unilateral frontoethmoidal encephalocele (an encephalocele at the junction of the frontal and ethmoidal bones), secondary to trauma sustained from an RTA several years prior to the current presentation. Few publications described recurrent meningitis related to acquired encephaloceles, such as that by Giunta et al., where a patient developed recurrent bacterial meningitis due to traumatic intranasal meningo-encephalocele [8]. A similar case was described by Kendirli et al., where a 14-year-old boy developed four episodes of pneumococcal meningitis after falling from a tree when he was nine years old. Imaging studies followed by transnasal endoscopic surgery revealed an osseous defect in the posterior ethmoidal sinus on the right side, where a dura mater sac was bulging into the sinus space; CSF was leaking into the nasal cavity as well [9]. The latter case most likely describes a meningocele, not an encephalocele, since there was no mention of brain tissue in the affected paranasal sinus.

Cases about acquired encephaloceles are mostly described in the form of case reports; to the best of our knowledge, there is very little, if any, on the incidence and prevalence of acquired encephaloceles. This also applies to the other forms of data, including complications and prognostic factors associated with encephaloceles, since most data presented are related to congenital forms. Karsonovich et al. listed that frontoethmoidal encephaloceles are associated with a better prognosis compared to occipital encephaloceles [7]. This is because occipital encephaloceles had higher incidents of complications - such as seizures and hydrocephalus - compared to frontal encephaloceles. Seizures occurred in around 17% of patients with occipital encephaloceles, but were low in the frontal encephaloceles. Hydrocephalus is four to five times more likely to happen in the occipital type relative to their counterparts [7].

The statistics related to recurrent meningitis are similar in different studies. Around 6% of meningitis cases became recurrent in several studies [10,11], and 5% in another study [5]. Mortality rate was around 20% in high-income countries [2,10]. Paradoxically, recurrent meningitis outcomes often surpass single-episode cases, primarily because recurrent patients recognize symptoms earlier and seek prompt intervention [10,12].

**Figure 6:**
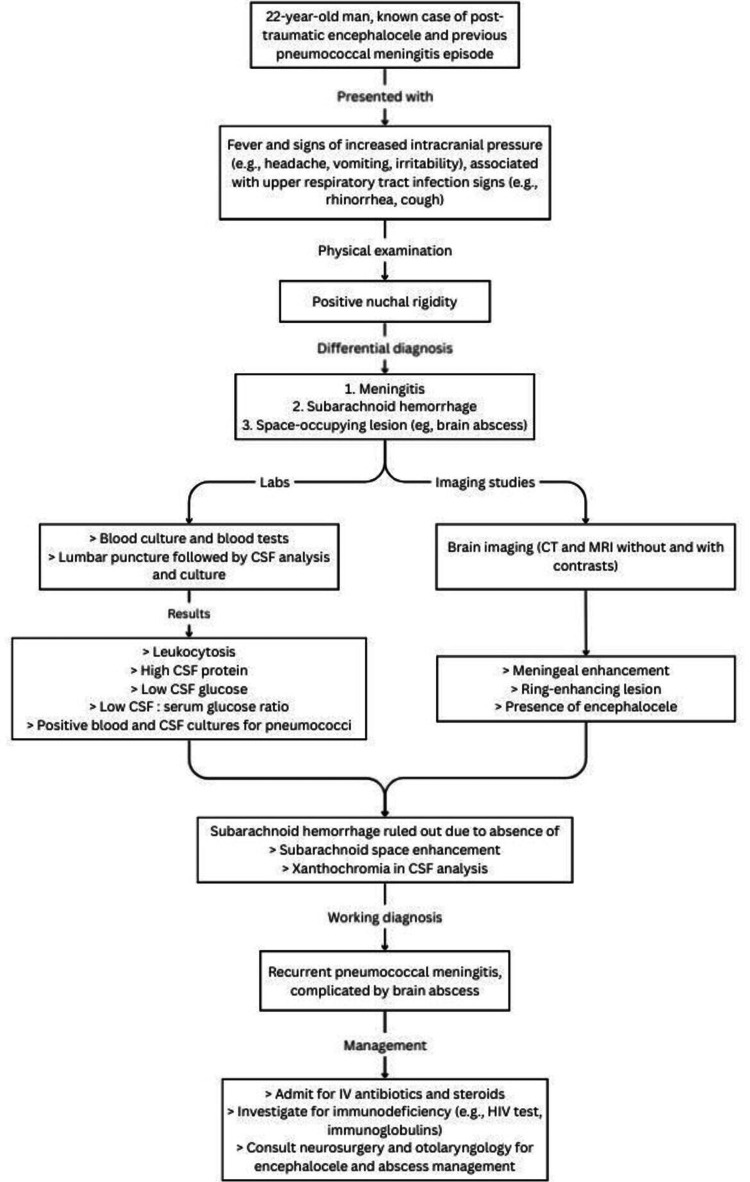
Flowchart demonstrating the diagnostic approach used in diagnosing and managing the patient’s condition.

In the present case, the patient presented with a group of signs and symptoms that included fever, headache, and vomiting, which are concerning for intracranial infections associated with increased intracranial pressure. Etiologies include meningitis, encephalitis, and space-occupying lesions (SOL), which include brain tumors, abscesses, or hemorrhages. Figure 6 shows a flowchart of the diagnostic approach used in this case.

Several case reports have documented bacterial meningitis occurring as a complication in patients who developed an acquired encephalocele after a head trauma event. The underlying pathophysiology has been reported as an abnormal communication between the central nervous system (which is usually covered by CSF) and the external environment. This allows pathogens to enter the central nervous system and cause an infection [6]. This is true for any anatomic anomaly in the cranium and spine, especially if it causes CSF leakage, manifesting as otorrhea and/or rhinorrhea [4,9]. Although these signs are strongly suggestive of CSF leakage, they may not always be present, as was with Kendirli et al. [9] and Sadr et al. [13] cases.

In all case studies mentioned earlier, meningitis was resolved after proper antibiotic therapy and surgical repair of the traumatic encephalocele [3,13-15]. The case report about the pediatric patient with recurrent meningitis due to traumatic meningocele did not experience meningitis after the surgical repair of the protruding dura mater [9]. It seems that the studies demonstrating meningitis in relation to an acquired encephalocele do not result in recurrent meningitis since the likely source of the infection, the encephalocele, has been corrected by surgical intervention. A case report, tracing back to 1991, demonstrated recurrent bacterial meningitis in the setting of an intranasal meningo-encephalocele post-trauma to the face [8].

The management of encephaloceles after the trauma event may be one of the reasons why acquired encephaloceles are poorly described in the literature. Underdiagnosis may be another element. Therefore, most of the data obtained about acquired encephaloceles are from case reports, which were used in the current research paper. Although there are papers that mention encephaloceles (which can be both congenital and acquired) are considered risk factors for recurrent meningitis [4,6,7,9], there are only a handful of documented cases of meningitis presented as recurrent. Thus, it would be appropriate to state that an underlying pathology (e.g., anatomical or immunological defects) should be considered when a patient has a positive history of, or presents with, recurrent meningitis.

Immunodeficiency is an important risk factor for recurrent meningitis [3]. In our case, the patient did not undergo an immunodeficiency workup because the patient did not have a history of recurrent infections or a history of serious infections, in the past (especially prior to his head trauma incident). Both meningitis episodes occurred after the traumatic head injury from the road traffic accident. The temporality of the events and the absence of recurrent and serious infections during early years of life and childhood make immunodeficiency an unlikely cause of meningitis in this case.

Surgical management is fundamental in the treatment of meningitis - whether single or recurrent episodes - associated with encephaloceles, regardless of whether congenital or acquired. The main priorities of the surgery are repairing the present defects and resecting nonfunctional neural tissue. Surgical options are typically open, but the endoscopic approach - one of the minimally invasive surgical approaches - may be used in certain conditions, such as sphenoid or ethmoidal encephaloceles [7]. Complications of encephalocele surgeries are numerous and range from facial deformities and incomplete repair to neurovascular structure injury, encephalocele recurrence, and, similar to other surgeries and procedures, infections (e.g., meningitis, encephalitis) [7].

## Conclusions

In patients presenting with recurrent meningitis, clinicians should evaluate for immunologic or infectious causes, and when these are absent or not suspected from the history, structural defects should also be considered. Given that anatomic and immunologic defects contribute largely to the etiologies of recurrent meningitis, both should be considered in future similar cases. Regardless of the etiology, recurrent meningitis should prompt early imaging and intervention; the latter includes managing the current meningitis episode and identifying the underlying cause, followed by treating it. The presence of anatomical deformities should be corrected by surgical means to resolve the abnormal communication between the brain and the external environment. Failure to do such interventions may result in complications such as recurrent infections and abscesses, which may negatively affect the patient’s quality of life. Ultimately, although most encephaloceles or structural anomalies are of a congenital variant, having detailed studies (e.g., observational studies about acquired encephaloceles) can enhance understanding of similar future presentations.
